# The Excess Winter Deaths Measure: Why Its Use Is Misleading for Public Health Understanding of Cold-related Health Impacts

**DOI:** 10.1097/EDE.0000000000000479

**Published:** 2016-03-17

**Authors:** Shakoor Hajat, Antonio Gasparrini

**Affiliations:** From the London School of Hygiene & Tropical Medicine, London, United Kingdom.

## Abstract

**Background:**

Excess winter deaths, the ratio between average daily deaths in December–March versus other months, is a measure commonly used by public health practitioners and analysts to assess health burdens associated with wintertime weather. We seek to demonstrate that this measure is fundamentally biased and can lead to misleading conclusions about health impacts associated with current and future winter climate.

**Methods:**

Time series regression analysis of 779,372 deaths from natural causes in London over 15 years (1 August 1997–31 July 2012),collapsed by day of death and linked to daily temperature values. The outcome measures were the excess winter deaths index, and daily and annual deaths attributable specifically to cold.

**Results:**

Most of the excess winter deaths are driven by cold: The excess winter deaths index decreased from 1.19 to 1.07 after excluding deaths attributable to low temperatures. Over 40% of cold-attributable deaths occurred outside of the December–March period, leading to bias in the excess winter deaths measure. Although there was no relationship between winter severity and annual excess winter deaths, there was a clear correlation with annual cold-attributable deaths.

**Conclusions:**

Excess winter deaths is not an appropriate indicator of cold-related health impacts, and its use should be discontinued. We advocate alternative measures. The findings we present bring into doubt previous claims that cold-related deaths in the UK will not reduce in future as a result of climate change.

Many parts of the world experience higher rates of mortality and morbidity during winter months compared with at other times of the year. Part of this wintertime excess can be attributed to seasonal infections such as influenza, but winter weather also plays a key role, particularly ambient temperature.^1^ Historically, the UK has experienced a high wintertime health burden which, on the face of it, is higher than many of its European neighbors.^2^ Although there is evidence that vulnerability to wintertime weather decreased during much of the past century,^3^ today thousands of preventable deaths still occur in the UK from cold weather each year and winter also puts annual pressures on the UK’s health-care services. Proper assessment of the health burdens associated with cold weather is important to inform both current public health policy and what may be expected in future due to climate change.

A common and simple way to quantify winter mortality impacts is to measure the number of deaths occurring during the winter season compared with other times of the year. The ratio of mortality between the selected seasons is considered an index where a value greater than one indicates a winter excess. The most common index is the excess winter deaths measure which considers the average number of daily deaths in the four coldest months of the year (December–March for northern hemisphere countries) compared with the average daily deaths in the preceding August–November and the following April–July, or similar variations.^4^ As noted, this excess is high in the UK compared with many other parts of Europe, including the colder Scandinavian countries.^5^ However, such comparisons are influenced by differences in the distribution of deaths across the winter—for example, in England, the cold winter period is more concentrated in the months used in the numerator of the index (December–March), whereas in Scandinavian countries, the cold period lasts longer.

There are also problems with interpreting such an index: the deaths due to cold that do fall outside of the December–March period will bias the excess winter deaths measure since, by definition, they contribute to the comparison months in the calculation. This bias can be substantial because many deaths attributable specifically to falls in temperature occur during even moderate winter conditions.^6^ Furthermore, the measure is very crude since it assumes that seasonal changes in health occur according to fixed calendar dates; a high excess winter deaths may also be caused by a flu epidemic or other seasonal factors unrelated to weather. The index can also be influenced by unusual mortality patterns at other times of the year used in the denominator (e.g., heat waves). The measure also does not allow for identification of which weather factors are important for ill health, or for the specific conditions that are adverse, for example, are cold-spells occurring early in the winter worse than later ones?^7^

Given these problems and inherent biases, excess winter deaths is of limited use in measuring the impacts of cold weather on public health. Its use for this purpose is somewhat entrenched due to the Office for National Statistics long-standing practice of releasing annual estimates of excess winter deaths in relation to each winter season.^8^ The confusion is further propagated by Public Health England’s Cold Weather Plan, which has a stated aim to reduce excess winter deaths in England,^9^ and by the development of quality standards by the National Institute for Health and Care Excellence to prevent excess winter deaths.^10^

Excess winter deaths is also regularly used by researchers.^2,11–17^ In many cases, this value is used as the outcome measure in the study of cold weather-related factors, such as fuel poverty, housing, and winter fuel payments,^18–22^ but with no consideration given as to how the use of excess winter deaths to represent cold-related health may have influenced findings. A good example of this is provided by an article in nature climate change that, having observed a lack of association between recent winter severity and excess winter deaths, mistakenly concludes that milder winters due to climate change will not reduce cold-related deaths in future.^23^

Here, we analyze daily and annual deaths in London over a 15-year period to quantify the contribution of ambient temperature to the excess winter deaths measure and demonstrate how analysis of excess winter deaths in past studies has led to inappropriate conclusions about cold-related health impacts associated with current and future climate. We seek to encourage use of alternative measures of wintertime health risk.

## METHODS

### Data

All deaths excluding deaths from external causes (ICD-10 codes S and above) in Greater London during August 1997–July 2012 were obtained from the Office for National Statistics and aggregated to create a time series of the daily number of deaths. No ethical review was required as only collapsed data were used. The start/end dates of the study period were chosen to coincide with the months used in the calculation of excess winter deaths. Mortality data more recent than 2012 were unavailable. To characterize complete winters, each year in analysis was defined as the period August–July rather than a calendar year.

Outdoor measurements of daily mean temperature were obtained from the British Atmospheric Data Centre (http://badc.nerc.ac.uk) for the same time period. Using a published algorithm,^24^ one representative temperature series for London was created by averaging data from all monitoring stations in the region that contained nonmissing values on at least 75% of days during the study period.

### Analysis

The association between season, temperature, and mortality was first characterized using time series regression analysis, following modeling choices described in recent publications.^6,25^ In brief, regression models assumed Poisson variation and allowed for overdispersion. In the first model (model 1), underlying seasonal patterns in the mortality series (unrelated to temperature) and any trends were controlled for using natural cubic splines of time with 8 degrees of freedom per year of data. Indicator terms were used to model day-of-week effects.

In the second model (model 2), further terms were added to model 1 to quantify the contribution of temperature to mortality. Distributed lag nonlinear models were used to describe the exposure–lag–response relationship with temperature, using a bidimensional spline with 5 × 5 degrees of freedom, extended up to 21 days of lag.^26^ This approach can flexibly capture nonlinear and delayed effects of temperature. The relationship between temperature and mortality was summarized as the exposure–response curve of relative risk accumulated across all lags. The number of deaths on each day of the series attributable to low temperatures was computed, using as reference and cutoff the minimum mortality temperature, which is the value of temperature at which mortality risk is lowest.^27^

Excess winter deaths was calculated by comparing the average daily deaths during the winter season (December–March) to the average number at other times of the year (preceding August–November and following April–July). This measure was computed in three ways: (1) using the observed number of daily deaths; (2) using the number of daily deaths predicted by model 1; and (3) using the number of daily deaths predicted by model 2, that is, once those deaths attributable to temperature were excluded.

Finally, to demonstrate the hazards of using excess winter deaths to represent health burdens associated with wintertime weather, the severity of each winter in the dataset was correlated against annual excess winter deaths, as was done in a previous study,^23^ but also against annual cold-specific attributable fractions estimated from the regression model above. To allow for a consistent comparison with the previous study, only the cold-attributable fraction associated with temperatures below 5°C was quantified for this component of the study. Analyses were conducted in R (version 3.2.3, 2015, Vienna) and STATA (version 13, 2013, TX).

## RESULTS

### Contribution of Temperature to Excess Winter Deaths

**FIGURE 1.**
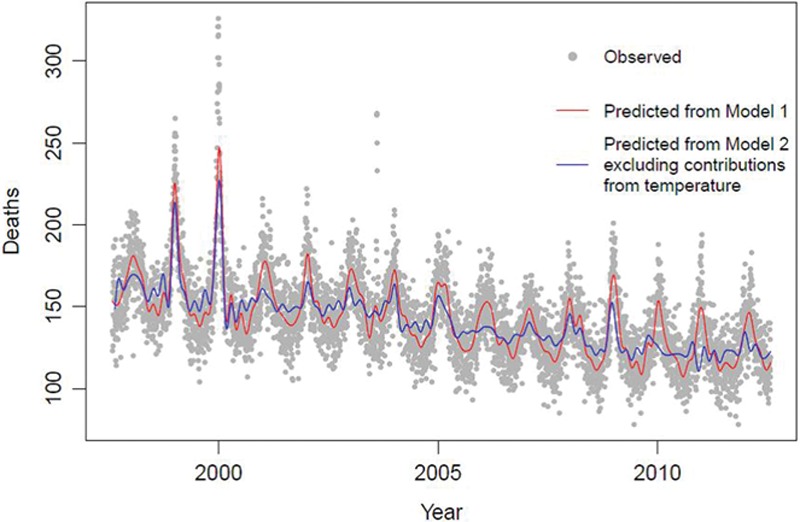
Daily mortality in London during 1997–2012, as daily deaths observed and predicted by model 1 (with no temperature control) and model 2 (with temperature control and exclusion of temperature-attributed deaths).

The excess winter deaths in London between 1997 and 2012, computed from observed mortality, was 1.19—indicating a 19% increase in deaths on days during the winter months (December–March) compared with other times of the year. This is consistent with the value obtained using mortality predicted from model 1 (with no control for temperature), where excess winter deaths of 1.19 is estimated (red line in Figure 1). However, once temperature-attributed deaths are excluded from the model (model 2) Excess winter deaths decreases substantially to 1.07 (blue line). This shows that the great majority of the wintertime excess is due to exposure to ambient cold temperature, with the remainder likely to be explained by influenza (especially in epidemic winters such as 1999/2000) and other seasonal factors.

### Cold-specific Deaths

**FIGURE 2.**
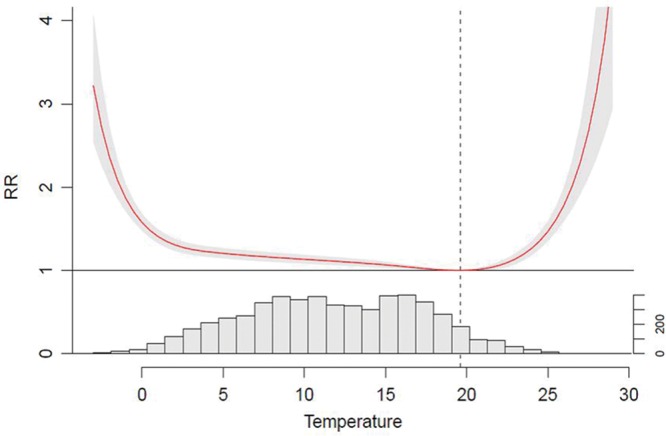
Exposure–response relationship between temperature and mortality accumulated over lag 0–21 days, and daily temperature distribution (frequency of days) in London during the period 1997–2012.

Figure 2 shows the seasonally adjusted relationship between ambient mean temperature (lags 0–21 days) and the RR of death in London. It shows that temperatures do not need to be particularly extreme before an increased risk in death is observed above and below the minimum mortality temperature of 19.6°C (dashed line in Figure 2). In terms of cold-related mortality, an increased risk at moderate temperatures is also observed which is important to note since moderate temperature days occur more often than more extreme temperature days. We estimate that about 11% of deaths from natural causes are attributable to all low temperatures, indicating that cold remains an important risk factor for mortality in recent years.

**FIGURE 3.**
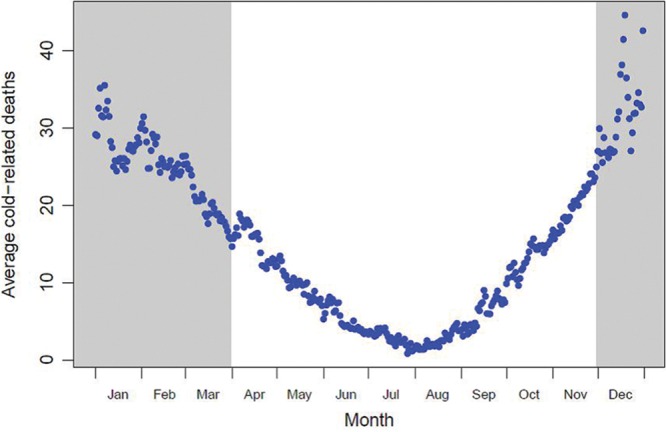
Average daily cold-attributed deaths by day of year in London.

Figure 3 shows the daily distribution of cold-attributable deaths in London averaged across the 15 years. Unsurprisingly, the highest counts are in the coldest months; however, the figure shows that a non-negligible number of cold-attributable deaths also occur outside of the December–March period. In a typical year, about 3,213 cold-attributable deaths occur during the December–March period; however, a further 2,257 cold deaths also occur at other times of the year.

### Annual Comparisons

**FIGURE 4.**
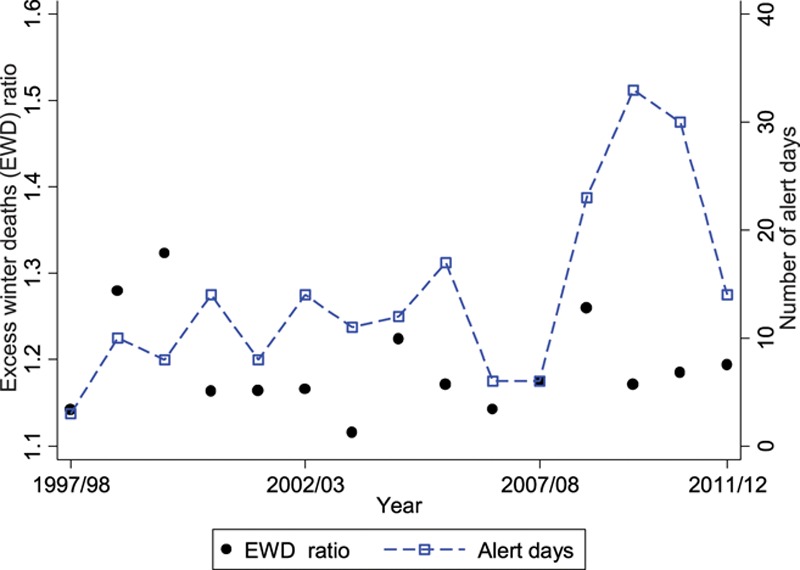
Annual excess winter deaths and winter severity (number of alert days) in London during 1997–2012.

Figure 4 shows the annual excess winter deaths ratio during 1997–2012 calculated from the observed data, as well as the annual number of days below 2°C as a measure of winter severity—this is the threshold used for a level 2 alert in the Public Health England Cold Weather Plan which runs from level 0 to level 4. The figure confirms that the excess winter deaths ratio has been fairly stable over the past 15 years, but with some high values in the winters of 1998/1999, 1999/2000, 2004/2005, and 2008/2009, but these do not coincide with cold winters.

**FIGURE 5.**
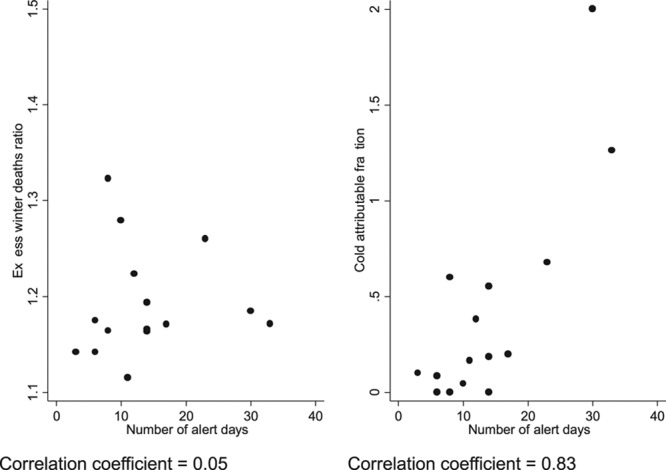
Correlation between annual winter severity (number of alert days) and excess winter deaths, and annual winter severity and cold-attributable fractions.

By contrast, when considering the cold-attributable fractions (below 5°C) stratified by year, the three highest estimates were observed in the winters of 2008/2009, 2009/2010, and 2010/2011, which were also the three coldest winters. Therefore, even though there is little relationship between winter temperatures and excess winter deaths in recent years, there is an association with deaths specifically attributable to cold. This is shown in Figure 5, which presents annual correlations of winter severity (alert days) with excess winter deaths, where no correlation is observed, and with the cold attributable fractions, where there is a clear correlation. Similar patterns are also observed when considering alternative measures of winter severity, such as average temperature during December–March (not shown).

## DISCUSSION

The analysis presented in this article is motivated by the need to convey to public health practitioners that excess winter deaths is not a good indicator of cold-related health burdens. Historically, annual excess winter deaths exhibited a correlation with winter severity in the UK because a general trend of decreasing excess winter deaths coincided with a period during which winters have become milder.^28^ In the past 15 years or so, however, the excess winter deaths measure has been relatively stable, so only data since 1997 were considered in this analysis. During this period, there is no annual correlation between excess winter deaths and winter weather. However, winter weather and specific cold impacts are highly correlated.

Although excess winter deaths is easy to calculate and is readily understood by the lay public and policymakers, the results presented here demonstrate that it is not a credible indicator of health burdens associated with wintertime weather. Indeed, its use for this purpose may be worse than having no information since its multiple biases obscure the true situation. For example, although about 3,213 cold-related deaths occur during December–March in a typical year, a further 2,257 cold deaths (over 40%) occur outside of this period. These additional cold deaths are contributing to the “wrong” side of the equation in the excess winter deaths calculation. Consequently, one can imagine a scenario where a particularly harsh winter that begins much earlier than December and/or ends much later than March could paradoxically lead to a *lower* excess winter deaths value since the additional deaths are all being counted in the comparison months.

Researchers who use excess winter deaths to draw conclusions about cold-related health can also be misled. Staddon et al.^23^ conclude that climate change will not result in a reduction in winter-related deaths in the UK because they observed no association between excess winter deaths and winter severity in recent years, only between the measure and flu. We demonstrate that the contribution of flu or any other seasonal factor to excess winter deaths is actually quite small in comparison to temperature. Had Staddon et al.^23^ eliminated the seasonal biases that excess winter deaths is subject to and instead characterized the specific relationship with weather, a strong association with winter severity would have been observed. Consequently, they would have reached the opposite conclusion to the one stated in their article. Climate change assessments for the UK that characterize specific cold risk all report that milder winters will lead to reduced cold-related health impacts.^29–33^ Nevertheless, cold-related health will remain a serious public health problem in the UK that policymakers will need to continue to address, especially given uncertainties in the direction of future winter climate.^34^ Furthermore, any expected reductions may be countered to some extent by future population growth and ageing.^31^

International comparisons are also more insightful when considering cold risk rather than excess winter deaths.^6^ The UK is regarded as having one of the highest burdens from cold weather in Europe because its excess winter deaths measure is high.^5^ However, country differences in the distribution of cold deaths and the biases this introduces into excess winter deaths may account for this. A previous comparison of cold-specific mortality risk across Europe found that vulnerability in London was not unusually high, with the RR being midrange within the 15 cities studied.^35^ But even this evidence does not provide a complete picture because the number of cold days experienced is not considered. Attributable fraction, which takes into account cold risk but also the number of days on which that risk is observed, is the most useful indicator of cold-related health burdens.^27^ This quantity can be usefully estimated even with 1 year of data for a large city such as London, and the Office for National Statistics should consider providing these instead of excess winter deaths for more reliable indication of health impacts associated with wintertime weather.

Our findings reveal that cold makes the greatest contribution to excess winter deaths, and polices aimed at reducing cold deaths further should be the priority for public health winter planning. The remaining excess is likely due to influenza and other seasonal factors, including possibly seasonal air pollutants. We did not consider the role of air pollution in our analysis; however, such a contribution is likely to be small in comparison to temperature, and it has been argued that pollution should not be a confounder of any temperature–health association.^36^

In conclusion, despite its inherent biases, excess winter deaths is commonly and unquestioningly used as an indicator of health impacts associated with winter weather. Its use should be discontinued and more appropriate measures adopted to quantify burdens associated with current and future winter climate.
